# Mesenchymal Stromal-Like Cells in the Glioma Microenvironment: What Are These Cells?

**DOI:** 10.3390/cancers12092628

**Published:** 2020-09-15

**Authors:** Anne Clavreul, Philippe Menei

**Affiliations:** 1 Département de Neurochirurgie, CHU, 49933 Angers, France; phmenei@chu-angers.fr; 2 Université d’Angers, CHU d’Angers, CRCINA, F-49000 Angers, France

**Keywords:** gliomas, mesenchymal stem cells, cancer-associated fibroblasts, microenvironment

## Abstract

**Simple Summary:**

We review here what is currently known about the role of mesenchymal stromal-like cells, which complicate our understanding of the glioma microenvironment. We provide an overview of the major studies on these cells, highlighting their role in tumor progression and prognosis. Researchers and clinicians should consider these cells to be an integral component of the glioma microenvironment, and of considerable potential value for future prognostic and therapeutic perspectives.

**Abstract:**

The glioma microenvironment is a critical regulator of tumor progression. It contains different cellular components such as blood vessels, immune cells, and neuroglial cells. It also contains non-cellular components, such as the extracellular matrix, extracellular vesicles, and cytokines, and has certain physicochemical properties, such as low pH, hypoxia, elevated interstitial pressure, and impaired perfusion. This review focuses on a particular type of cells recently identified in the glioma microenvironment: glioma-associated stromal cells (GASCs). This is just one of a number of names given to these mesenchymal stromal-like cells, which have phenotypic and functional properties similar to those of mesenchymal stem cells and cancer-associated fibroblasts. Their close proximity to blood vessels may provide a permissive environment, facilitating angiogenesis, invasion, and tumor growth. Additional studies are required to characterize these cells further and to analyze their role in tumor resistance and recurrence.

## 1. Introduction

Blood vessels, immune cells, and neuroglial cells are classically considered to be the major cellular components of the microenvironment of gliomas [1], but there is recent evidence to suggest that gliomas also harbor stromal cells with the characteristics of mesenchymal stem cells (MSCs) and cancer-associated fibroblasts (CAFs). These stromal cells have been given various names in previous studies: glioma-associated human MSCs (GA-hMSCs) [2,3,4], glioma-associated MSCs or glioblastoma-derived MSCs (gbMSCs) [5,6], glioma stroma-MSCs (GS-MSCs) [7], brain tumor-derived MSCs (BT-MSCs) [8], mesenchymal stem-like cells (MSLCs) [9,10,11], tumor MSCLs (tMSLCs) [12,13,14,15], glioma stromal MSLCs (GS-MSLCs) [16], CAF-like cells [17], glioblastoma-associated stromal cells or glioma-associated stem cells (GASCs) [18,19,20,21,22,23,24,25,26]. Here, we will call them GASCs (glioma-associated stromal cells), and we provide an overview of the principal studies on these cells to date.

## 2. General Characteristics of GASCs

GASCs have been isolated from primary cultures of cells from mouse orthotopic transplantation tumor models [7,8], human low-grade gliomas (LGGs), human high-grade gliomas (HGGs) [2,3,4,5,6,8,9,10,11,12,13,15,18,19,20,26], and the peritumoral region of glioblastomas (GBs) [22,23,24,25] (Table 1). Cultured GASCs are spindle-shaped, adherent in culture, can be subcultured many times, and express the mesenchymal surface markers CD73, CD90, and CD105. By contrast, they do not express endothelial cells (CD31), macrophage (CD14), astrocyte (GFAP), and hematopoietic stem cell (CD34, CD45) markers. The expression of markers associated with CAFs, such as fibroblast-specific protein 1 (FSP1/S100A4), alpha-smooth muscle actin (α-SMA), and platelet-derived growth factor receptor-beta (PDGFRβ) has also been reported. In general, GASCs have the potential to differentiate into three types of cell—adipocytes, chondrocytes, and osteocytes—and are nontumorigenic (Table 1).

It is difficult to identify GASCs in situ due to the lack of specific cell surface antigens. In some studies, they have been identified by immunofluorescence labeling on sections of human glioma surgical specimens, using MSC markers, such as CD105 and PDGFRβ, or markers typically expressed by CAFs, such as FSP1/S100A4, α-SMA, and the fibroblast and mesodermal cell marker TE-7 (Table 1) [3,9,17,22]. These studies showed GASCs to be localized predominantly around blood vessels, but also in the tumor tissue proper. Other studies were based on flow cytometry, with analyses of the co-expression of the MSC markers CD105, CD73, and CD90 on fresh surgical glioma specimens [3,4]. These studies showed that the fraction of triple-positive cells varied between specimens, ranging from 0.7% to 19.5%.

## 3. Origin of GASCs

The origin of GASCs has yet to be determined. Hossain et al. [3] suggested that GASCs may differentiate from glioma stem cells (GSCs). This hypothesis is consistent with reports showing that GSCs can differentiate into stromal cells, including endothelial cells in particular [27]. However, most GASCs are genetically different from GSCs. They are typically diploid and do not harbor the genetic alterations commonly seen in GSCs, such as losses of chromosome 10 or gains of chromosome 7, suggesting that GASCs are probably recruited to the tumor from a source other than GSCs [3,22]. GASCs may be derived from an epithelial-to-mesenchymal like transition of reactive astrocytes which acquire stem cell properties [20]. These reactive astrocytes may originate from local astrocytes or from the migration and differentiation of neural progenitor cells found in the subventricular zone [28]. Alternatively, as suggested for CAFs, GASCs may arise from the transdifferentiation of pericytes and vascular smooth muscle cells, and from endothelial cells through the process of endothelial-to-mesenchymal transition [29]. GASCs can also originate from MSCs. This latter hypothesis is supported by many studies showing the migration of MSCs isolated from different sources such as the bone marrow towards established gliomas and their integration into the tumor vasculature [30,31,32]. The mechanism underlying the tropism of MSCs for gliomas has not been fully elucidated, but numerous chemotactic factors have been implicated in this process, including VEGF, SDF-1/CXCL12, and IL-8 [33,34,35]. Cells similar to MSCs in terms of in vitro growth, surface markers, and trilineage mesenchymal differentiation have also been isolated from normal brains [36,37,38]. Conflicting results have been reported, but several studies have shown that MSCs promote tumor progression [30,39].

## 4. Functions of GASCs

The interaction between “naïve” MSCs and tumor cells remains poorly understood [30] but experimental evidence in favor of a tumor growth-enhancing role of GASCs has been obtained. We found that GASCs isolated from the peritumoral region of GBs had tumor-promoting effects on human GB cell lines in vitro and in vivo [22]. The subcutaneous injection of these cells, together with U87MG GB cells, into nude mice resulted in the induction of tumors larger than those induced by the injection of U87MG cells alone or together with control stromal cells obtained from non-GB peripheral brain tissues [22]. Other studies have shown that GASCs isolated from fresh human glioma specimens can promote the proliferation and self-renewal of tumor-initiating GSCs in vitro, and enhance GSC tumorigenicity in intracranial models in vivo [3,16]. These effects may be mediated by both the secretion of soluble growth-promoting factors, such as IL-6, and the exosomal delivery of specific oncogenic miRNAs [2,3,19].

GASCs also have angiogenic properties. A comparison of GASCs from the GB peritumoral zone with control stromal cells derived from non-GB peripheral brain tissues, based on iTRAQ labeling and mass spectrometry, showed that GASCs overproduced several proteins involved in the promotion of tumor angiogenesis or in blood vessel development, including CSPG4/NG2, CRYAB, CNN1, CALD1, and VASP [23]. Furthermore, the secretion of angiogenesis factors, such as SDF-1/CXCL12, and HGF, was upregulated in GASCs. Consistent with the overproduction of these proteins by GASCs, the inoculation of nude mouse striatum with both these cells and U87MG cells promoted angiogenesis, leading to an increase in the number of small vessels [23]. Similarly, Kong et al. [16] observed enhanced microvessel formation in mice receiving injections of GSCs cocultured with GASCs relative to mice receiving injections of GSCs cultured alone. GB-conditioned medium may also induce GASC differentiation into pericytes and enhance the attachment of these cells to tube-like vessels formed by human umbilical vein endothelial cells on Matrigel, stabilizing capillary-like structures in vitro [5]. Two subpopulations of GASCs differing in terms of CD90 expression were recently sorted from fresh glioma tissues (WHO II–IV gliomas) [6,11]. Both in vivo and in vitro experiments have shown that CD90^high^ GASCs significantly promote glioma cell growth, and that CD90^low^ GASCs promote angiogenesis via pericyte transition [6].

GASCs have also been shown to boost the invasiveness of GB cells [10,12,13,17,23,25]. Force-mediated ECM remodeling through CCL2/JAK1/MLC2 signaling and the secretion of CXCL14 and C5a may be drivers of the glioma invasion mediated by GASCs [10,12,13,25].

## 5. Heterogeneity of GASCs

Taghipour et al. [14] found that GASCs isolated from newly diagnosed LGGs and HGGs had different proteomic profiles. Proteins associated with mesenchymal cells (vimentin and transglin), and with tumor aggressiveness with potential secretory behavior (e.g., cathepsin B) were among proteins for which differential expression was observed. GASCs isolated from LGGs and HGGs also differed in terms of cancer cell adhesion [18]. The adhesion strength between GASCs and GSCs from HGGs was significantly lower than that observed between GASCs and GSCs from LGGs. In addition to the differences between the GASCs of LGGs and HGGs, GASCs may also differ between gliomas with the same histopathological classification. As indicated above, two subpopulations of GASCs differing in terms of CD90 expression have been sorted from the same glioma tissues, with CD90^low^ GASCs more abundant than CD90^high^ GASCs [6,11]. We have shown that by analyzing the transcriptome and methylome of GASCs from GB-free surgical margins and control stromal cells derived from non-GB peripheral brain tissues, that two surgical margin microenvironments can be encountered in GB patients: a surgical margin microenvironment containing GASCs with procarcinogenic properties, and another containing GASCs without such properties [24]. Thus, the genetic background of tumor cells may be a key determinant of the GASC-tumor relationship. Consistent with this hypothesis, we observed that the migration pattern of MSCs and their effect on glioma cell growth depended on the tumor cell line used [40]. Similarly, Breznik et al. [41] demonstrated that MSC/GB crosstalk differed between two established GB cell lines, U87 and U373, with MSCs inhibiting invasion by U87 cells but enhancing that by U373 cells. Differential gene expression between U87 and U373 cells may account for the different responses of these cell lines to MSCs.

## 6. GASCs: A Prognostic Marker for Gliomas

The deletion of both chromosome 1p and 19q, O-methylguanine DNA methyltransferase (MGMT) promoter methylation, and mutations of the isocitrate dehydrogenase (IDH) 1 and 2 genes are the principal prognostic factors for gliomas identified to date [42,43]. Several studies have reported that GASCs may be another prognostic factor for gliomas. For example, Bourkoula et al. [19] defined a GASC score for predicting the prognosis of human LGGs. This score was based on the levels of nine surface proteins: three stem cell antigens (CD271, CD133, and ABCG2), three adhesion proteins (CD49a, CD49d, and E-Cadherin), and three mesenchymal markers (CD90, CD73, and CD105). Specifically, GASCs obtained from LGG patients with poor prognosis were characterized by higher levels of stem cell-related markers, integrin downregulation, and a variable modulation of mesenchymal markers. Moreover, in multivariate analysis with various covariates, including IDH mutations, 1p/19q co-deletions, and MGMT promoter methylation, GASC score was the only independent predictor of overall survival (OS) and malignant progression-free survival (PFS) for LGG patients. Ius et al. [26] used next-generation sequencing to compare the gene expression profiles of GASCs isolated from LGGs with a good prognosis, and GASCs isolated from LGGs undergoing rapid anaplastic transformation. They identified an NF-κB signature composed of 14 genes that was able to predict OS in a dataset for 530 newly diagnosed patients with diffuse LGG from The Cancer Genome Atlas (TCGA). Moreover, the levels of the NF-kB-p65 protein in the nucleus, assessed by an immunohistochemical method, were found to be an independent predictor of both OS and malignant PFS in 146 grade II LGG patients. He et al. [25] showed that CXCL14 is overexpressed in GASCs and predicts clinical outcome. They found that CXCL14 expression was negatively correlated with OS in patients with glioma in the TCGA dataset. Yoon et al. (2016) [15] were able to isolate GASCs from 58.5% of patients with GB via primary culture. They found that the GB patients from whom they were able to isolate GASCs had poorer survival than those from whom no GASCs were isolated. Similarly, Shahar et al. [4] analyzed GASCs from surgical HGG specimens, based on coexpression of the MSC markers CD105, CD73, and CD90, and determined the fraction of triple-positive cells in the fresh tumor mass or in cultured tumors. They found that the percentage of GASCs in gliomas was variable between tumors and that patients with high percentages of GASCs in their tumors (fresh or cultured) had a poorer OS than those with a low percentage of these cells. All these data highlight the potential utility of GASCs as a prognostic marker for human LGGs and HGGs.

## 7. GASCs and Cellular Models

The development of new models of human glioma providing new insight into tumor biology and improving the prediction of response to treatment in patients is challenging. GSCs are difficult to isolate from LGGs. Ius et al. [26] showed that patient-derived GASCs could potentially be used instead, for the identification of novel LGG prognostic/predictive biomarkers. As described above, they demonstrated the potential of an NF-κB signature extrapolated from their GASC study for predicting LGG prognosis. Methods for constructing self-organizing three-dimensional (3D) coculture systems, known as organoids, have been developed, to mimic in vivo tumors. Hermida et al. [44] described 3D bioprinting methods using bioinks based on a modified alginate for the preparation of tumor models incorporating tumor and stromal cells from GB. They observed that the bioprinting of GSCs, together with both patient-derived GASCs and human microglia, had no adverse effect on the viability of these cell types. The use of such 3D-bioprinted GB cell constructs is promising for the future preclinical drug sensitivity testing and studies of the tumor microenvironment.

## 8. GASCs: A Future Treatment Target or Therapeutic Tool?

Given the tumor growth-enhancing role of GASCs, these cells are promising new targets for glioma treatment. However, additional studies are required to identify candidate target molecules. We recently turned this problem around by using the properties of these cells to trap tumor cells, rather than targeting them directly [45]. The concept of tumor cell traps, which emerged from the ecological trap strategy, was developed for brain tumors as a means of attracting the residual cancer cells surrounding the surgical cavity to the cavity, where they are trapped within a biomaterial support that can be targeted by treatment, such as stereotactic radiosurgery [46,47]. As indicated above, we and others have shown that GASCs increase the invasiveness of GB cells [10,12,13,17,24,25]. Based on this knowledge, we used GASC-conditioned medium as a source of chemoattractants to load the trap. We chose a bacterial cellulose (BC) scaffold as the trap matrix, because cellulose-based materials are already used in clinical practice, as exemplified by Surgicel^®^, which is widely used in neurosurgery due to its hemostatic effects and high tissue compatibility. BC is also highly flexible, and is therefore easy to introduce into the tumor bed after resection, and its visibility on MRI may facilitate stereotactic radiosurgery. We found that the structure of BC membranes, with their random assembly of nanofibrils, was ideal for the trapping of tumor cells, which, once attached to the surface of the membrane, were unable to move, pass through the membrane or escape, even in the presence of an attractive medium in close proximity [45]. We also demonstrated that BC membranes loaded with GASC-conditioned medium could release and attract tumor cells in vitro [45]. Advanced methods should be developed to transform this trap into a chemotaxis device for the diffusion of chemoattractants over large distances and at high enough concentrations to establish a concentration gradient extending into the surrounding environment, for the trapping of GB cells infiltrating tissues several centimeters away from the resection cavity.

## 9. Conclusions

**Figure 1:**
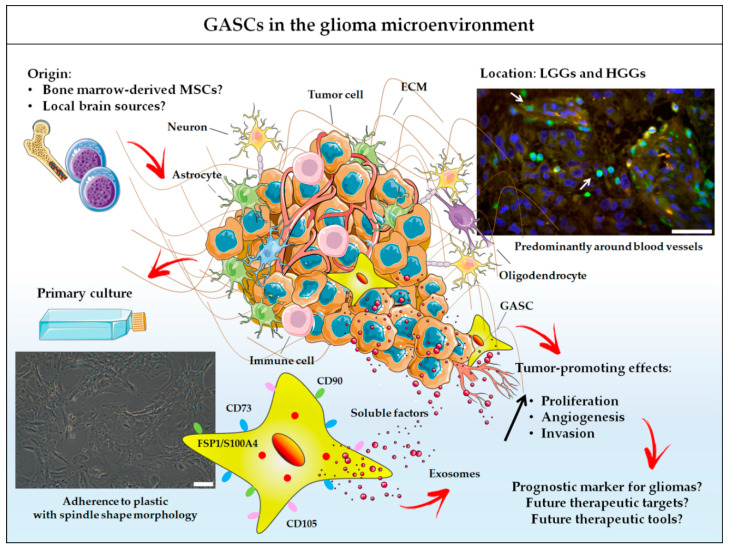
GASCs in the glioma microenvironment. GASCs can now be considered an integral component of the microenvironment of human LGGs and HGGs, alongside blood vessels, immune cells and neuroglial cells. They may be recruited from local brain sources or from the bone marrow, and are mostly found around blood vessels: arrows indicate S100A4^+^ cells in a GB region; scale bar = 50 μm. Cultured GASCs have properties similar to those of MSCs, such as adherence to plastic (scale bar = 100 µm), expression of surface antigens characteristic of MSCs (CD73, CD90, CD105), mesenchymal differentiation potential and a lack of tumorigenesis potential. They also have phenotypic and functional properties in common with the CAFs described in the stroma of carcinomas. In particular, GASCs express markers associated with CAFs, including FSP1/S100A4, and have tumor-promoting effects mediated by the secretion of soluble factors and exosomes. These cells are of potential interest for prognostic and therapeutic applications.

All these data indicate that GASCs are important stromal cells in the microenvironment of gliomas that should not be ignored (Figure 1). Their close proximity to blood vessels may provide a permissive environment, facilitating angiogenesis, invasion and tumor growth. Additional studies on the impact of GASCs on the response to treatments for gliomas, such as radiotherapy, chemotherapy, anti-angiogenic therapy, and laser interstitial therapy, are urgently required.

## Figures and Tables

**Table 1:** Characteristics and functions of GASCs.

Nomenclature Used	Sources	Method of Identification	Characteristics of Cultured GASCs	Major Characteristics or Functions of GASCs Identified	References
GS-MSCs	Orthotopic xenografting of human gCSCs in mice	- Primary cultures	- Spindle-shape morphology - Markers: Sca-1^+^, CD9^+^ CD45^−^, CD11b^−^, CD31^−^, NG2^−^ - Differentiation into adipocytes, osteoblasts and chondrocytes - Non-tumorigenic behavior	**Location of GASCs:** - Predominantly located around blood vessels.	[7]
GASCs	Peritumoral region from GBs	- Primary cultures - IF analysis: (FSP1/S100A4^+^)	- Adherence to plastic with spindle-shape morphology - Markers: α-SMA^+^, PDGFRβ^+^, FSP1/S100A4^+^, CD105^+^, CD73^+^, CD90^+^, CD31^−^, CD14^−^, GFAP^−^, CD34^−^, CD45^−^ - GASCs underwent osteogenesis, but not adipogenesis - Non-tumorigenic behavior - Diploid cells	**Tumor-supporting function:** - GASCs have tumor-promoting effects on glioma cell lines in vitro and in vivo. - GASCs increase angiogenesis in the orthotopic U87MG glioma model. **Heterogeneity of GASCs:** - Two subtypes of GASCs identified in surgical margins of GB patients: GASC-B promoted the development of tumors and endothelium, whereas GASC-A did not. **Invasion function:** GASC-secreted CXCL14 may drive glycolysis and cell invasion in glioma via the UCA1/miR-182/PFKFB2 axis. **Prognostic role:** CXCL14 is overexpressed in GASCs and predicts clinical outcome.	[21,22,23,24,25]
MSLCs/GS-MSLCs/tMSLCs	Human glioma specimens	- Primary cultures - IF analysis (CD105^+^/CD31^−^ or CD105^+^/NG2^−^)	- Adherence to plastic with spindle-shape morphology - Markers: CD105^+^, CD73^+^, CD90^+^, CD45^−^, CD31^−^, NG2^−^ - Differentiation into adipocytes, osteoblasts and chondrocytes - Non-tumorigenic behavior	**Invasion function:** - GASCs contribute to the abundance of HA in TME through HAS2 induction, thereby increasing the invasiveness of GB cells. - GASCs are involved in force-mediated proinvasive ECM remodeling through CCL2/JAK1/MLC2 signaling in GB, like CAFs in carcinoma. - GASCs promote the invasion of GB cells through the secretion of C5a into the TME, further increasing ZEB1 expression in GB cells via the C5aR1/p38 MAPK signaling pathway. **Prognostic role:** - The isolation of GASCs from the specimen of primary GB is negatively associated with patient survival.	[9,10,12,13,15,16]
BT-MSCs	- GL261 murine glioma model - Human GB specimens	- Primary cultures	Murine model: - Fibroblastoid morphology - Markers: Lin-Sca1^+^/CD9^+^/CD44^+^/CD166^+/−^ - Multilineage differentiation capacity - Tumorigenic behavior Human model: - Markers: CD44^+^, CD9^+^, CD166^+^	**Tumor-supporting function:** - The infiltration of GASCs is correlated with tumor progression. - GASCs increased the proliferation rate of GL261 cells in vitro.	[8]
CAF-like cells	Human GB specimens	- IF analysis: (α-SMA^+^/GFAP^-^ or TE-7^+^/GFAP^-^)	- Markers: α-SMA^+^, TE-7^+^, GFAP^−^	**Location of GASCs:** - Predominantly localized around abnormal blood vessels.	[17]
GA-hMSCs	Human glioma specimens	- Primary cultures - IF analysis: (CD105^+^/CD31^−^ or CD105^+^/PDGFRβ^+^) - Flow cytometry analysis: triple-positive (CD105^+^/CD73^+^/CD90^+^) cells	- Adherence to plastic with spindle-shape morphology - Markers: CD105^+^, CD73^+^, CD90^+^, CD45^−^, CD34^−^ - Did not harbor mutations common to GB - Differentiation into adipocytes, osteoblasts and chondrocytes (NB: some specimens differentiated into only two mesenchymal phenotypes) - Non-tumorigenic behavior	**Tumor-supporting function:** - Increase the proliferation and self-renewal of GSCs in vitro and enhance GSC tumorigenicity and mesenchymal features in vivo. - These effects are mediated by the secretion of soluble growth-promoting factors, such as IL-6, and by the exosomal delivery of specific oncogenic miRNAs, such as miR-1587. **Prognostic role:** - The percentage of GASCs is inversely correlated with OS.	[2,3,4]
MSLCs	Human glioma specimens	- Primary cultures	- Adherence to plastic with spindle-shape morphology - Markers: CD73^+^, CD105^+^, CD90^+^ or CD90^−^ - Differentiation into osteoblasts and, to some extent, adipocytes and chondrocytes	**Heterogeneity of GASCs:** - Two different subsets of GASCs, differing in their expression of the CD90 surface marker, were discovered after cell sorting. - The CD90^−^ GASCs produce more VEGF and PGE2 than their CD90^+^ counterparts.	[11]
tMSCs	Human glioma specimens	- Primary cultures	- Adherence to plastic with spindle-shape morphology - Markers: CD44^+^, CD105^+^, CD166^+^, CD45^−^, CD34^−^	**Heterogeneity of GASCs:** - GASCs derived from LGGs and HGGs have different proteome profiles. - Molecules associated with mesenchymal cells (vimentin and transglin), and tumor aggressiveness with potential secretory behavior (e.g., cathepsin B) were among those for which differential gene expression was detected.	[14]
GbMSCs	Human glioma specimens	- Primary cultures	- Adherence to plastic with spindle-shape morphology - Markers: CD73^+^, CD105^+^, CD44^+^, CD90^high^ or CD90^low^, CD31^−^, CD34^−^, CD14^−^, NG2^−^, PDGFRβ^−^ - Differentiation into adipocytes, osteoblasts, and chondrocytes	**Heterogeneity of GASCs:** - Two GASC subpopulations based on CD90 expression (CD90^high^ and CD90^low^). **Tumor-supporting function:** - CD90^high^ GASCs significantly promoted glioma cell growth whereas CD90^low^ GASCs promoted angiogenesis via pericyte transition.	[5,6]
GASCs	Human glioma specimens	- Primary cultures	- Adherence to plastic with spindle-shape morphology - Markers: CD105^+^, CD73^+^, CD90^+^, CD45^-^ - Differentiation into mesodermal derivatives, such as endothelial-, osteoblast-, and myocyte-like cells - Non-tumorigenic behavior	**Tumor-supporting function:** - GASCs support the malignant properties of both GB cell lines (A172 and U87) and human GSCs, mainly through the release of exosomes. - Exosomes released both from HGGs and LGGs were able to increase the in vitro aggressiveness of GB cells, although those from LGGs did so to a significantly lesser extent. **Invasion function:** - The strength of GSCs adhesion to GASCs appears to be significantly lower for cells derived from HGGs than for those derived from LGGs. - GSCs from HGGs firmly adhere to GASCs from LGGs, but not to those from HGGs. **Prognostic role:** - Ability of a score based on the expression of nine GASC surface markers (CD133, CD271, ABCG2, E-Cadherin, CD90, CD49a, CD49d, CD105, CD73) to predict OS and malignant PFS in LGG patients. - An NF-κB signature extrapolated from GASC predicts LGG prognosis.	[18,19,20,26]

BT-MSCs, brain tumor-derived MSCs; CAF-like cells, cancer associated fibroblast-like cells; GA-hMSCs, glioma-associated human MSCs; GASCs, glioblastoma-associated stromal cells or glioma-associated stem cells; GB, glioblastoma; GbMSCs, glioma-associated MSCs or glioblastoma-derived MSCs; gCSCs, glioma cancer stem cells; GSCs, glioma stem cells; GS-MSCs, glioma stroma-MSCs; GS-MSLCs, glioma stromal MSLCs; HA, hyaluronic acid; HAS2, HA synthase-2; HGGs, high grade gliomas; LGGs, low grade gliomas; MSCs, mesenchymal stem cells; MSLCs, mesenchymal stem-like cells; TME, tumor microenvironment; tMSCs, tumor MSCs; tMSLCs, tumor MSLCs; OS, overall survival; PFS, progression-free survival.

## References

[1] Quail D.F., Joyce J.A. (2017). The microenvironmental landscape of brain tumors. Cancer Cell.

[2] Figueroa J., Phillips L.M., Shahar T., Hossain A., Gumin J., Kim H., Bean A.J., Calin G.A., Fueyo J., Walters E.T. (2017). Exosomes from glioma-associated mesenchymal stem cells increase the tumorigenicity of glioma stem-like cells via transfer of miR-1587. Cancer Res..

[3] Hossain A., Gumin J., Gao F., Figueroa J., Shinojima N., Takezaki T., Priebe W., Villarreal D., Kang S.-G., Joyce C. (2015). Mesenchymal stem cells isolated from human gliomas increase proliferation and maintain stemness of glioma stem cells through the IL-6/gp130/STAT3 pathway. Stem Cells Dayt. Ohio.

[4] Shahar T., Rozovski U., Hess K.R., Hossain A., Gumin J., Gao F., Fuller G.N., Goodman L., Sulman E.P., Lang F.F. (2017). Percentage of mesenchymal stem cells in high-grade glioma tumor samples correlates with patient survival. Neuro Oncol..

[5] Yi D., Xiang W., Zhang Q., Cen Y., Su Q., Zhang F., Lu Y., Zhao H., Fu P. (2018). Human glioblastoma-derived mesenchymal stem cell to pericytes transition and angiogenic capacity in glioblastoma microenvironment. Cell. Physiol. Biochem. Int. J. Exp. Cell. Physiol. Biochem. Pharmacol..

[6] Zhang Q., Yi D.-Y., Xue B.-Z., Wen W.-W., Lu Y.-P., Abdelmaksou A., Sun M.-X., Yuan D.-T., Zhao H.-Y., Xiong N.-X. (2018). CD90 determined two subpopulations of glioma-associated mesenchymal stem cells with different roles in tumour progression. Cell Death Dis..

[7] Kim S.-M., Kang S.-G., Park N.-R., Mok H.-S., Huh Y.-M., Lee S.-J., Jeun S.-S., Hong Y.-K., Park C.-K., Lang F.F. (2011). Presence of glioma stroma mesenchymal stem cells in a murine orthotopic glioma model. Childs Nerv. Syst. ChNS Off. J. Int. Soc. Pediatr. Neurosurg..

[8] Behnan J., Isakson P., Joel M., Cilio C., Langmoen I.A., Vik-Mo E.O., Badn W. (2014). Recruited brain tumor-derived mesenchymal stem cells contribute to brain tumor progression. Stem Cells Dayt. Ohio.

[9] Kim Y.G., Jeon S., Sin G.-Y., Shim J.-K., Kim B.-K., Shin H.-J., Lee J.-H., Huh Y.-M., Lee S.-J., Kim E.-H. (2013). Existence of glioma stroma mesenchymal stemlike cells in Korean glioma specimens. Childs Nerv. Syst. ChNS Off. J. Int. Soc. Pediatr. Neurosurg..

[10] Lim E.-J., Kim S., Oh Y., Suh Y., Kaushik N., Lee J.-H., Lee H.-J., Kim M.-J., Park M.-J., Kim R.-K. (2020). Crosstalk between GBM cells and mesenchymal stem-like cells promotes the invasiveness of GBM through the C5a/p38/ZEB1 axis. Neuro Oncol..

[11] Svensson A., Ramos-Moreno T., Eberstål S., Scheding S., Bengzon J. (2017). Identification of two distinct mesenchymal stromal cell populations in human malignant glioma. J. Neurooncol..

[12] Lim E.-J., Suh Y., Yoo K.-C., Lee J.-H., Kim I.-G., Kim M.-J., Chang J.H., Kang S.-G., Lee S.-J. (2017). Tumor-associated mesenchymal stem-like cells provide extracellular signaling cue for invasiveness of glioblastoma cells. Oncotarget.

[13] Lim E.-J., Suh Y., Kim S., Kang S.-G., Lee S.-J. (2018). Force-mediated proinvasive matrix remodeling driven by tumor-associated mesenchymal stem-like cells in glioblastoma. BMB Rep..

[14] Taghipour M., Omidvar A., Razmkhah M., Ghaderi A., Mojtahedi Z. (2017). Comparative proteomic analysis of tumor mesenchymal-like stem cells derived from high grade versus low grade gliomas. Cell J..

[15] Yoon S.-J., Shim J.-K., Chang J.H., Moon J.H., Roh T.-H., Sung K.S., Lee J.-H., Kim E.-H., Kim S.H., Hong Y.-K. (2016). Tumor mesenchymal stem-like cell as a prognostic marker in primary glioblastoma. Stem Cells Int..

[16] Kong B.H., Shin H.-D., Kim S.-H., Mok H.-S., Shim J.-K., Lee J.-H., Shin H.-J., Huh Y.-M., Kim E.-H., Park E.-K. (2013). Increased in vivo angiogenic effect of glioma stromal mesenchymal stem-like cells on glioma cancer stem cells from patients with glioblastoma. Int. J. Oncol..

[17] Trylcova J., Busek P., Smetana K., Balaziova E., Dvorankova B., Mifkova A., Sedo A. (2015). Effect of cancer-associated fibroblasts on the migration of glioma cells in vitro. Tumour Biol. J. Int. Soc. Oncodev. Biol. Med..

[18] Andolfi L., Bourkoula E., Migliorini E., Palma A., Pucer A., Skrap M., Scoles G., Beltrami A.P., Cesselli D., Lazzarino M. (2014). Investigation of adhesion and mechanical properties of human glioma cells by single cell force spectroscopy and atomic force microscopy. PLoS ONE.

[19] Bourkoula E., Mangoni D., Ius T., Pucer A., Isola M., Musiello D., Marzinotto S., Toffoletto B., Sorrentino M., Palma A. (2014). Glioma-associated stem cells: A novel class of tumor-supporting cells able to predict prognosis of human low-grade gliomas. Stem Cells Dayt. Ohio.

[20] Caponnetto F., Beltrami A.P., Ius T., Skrap M., Cesselli D., Duffau H. (2017). Diffuse Low-Grade Glioma Associated Stem Cells. Diffuse Low-Grade Gliomas in Adults.

[21] Chen C., Sun C., Tang D., Yang G., Zhou X., Wang D. (2016). Identification of key genes in glioblastoma-associated stromal cells using bioinformatics analysis. Oncol. Lett..

[22] Clavreul A., Etcheverry A., Chassevent A., Quillien V., Avril T., Jourdan M.-L., Michalak S., François P., Carré J.-L., Mosser J. (2012). Isolation of a new cell population in the glioblastoma microenvironment. J. Neurooncol..

[23] Clavreul A., Guette C., Faguer R., Tétaud C., Boissard A., Lemaire L., Rousseau A., Avril T., Henry C., Coqueret O. (2014). Glioblastoma-associated stromal cells (GASCs) from histologically normal surgical margins have a myofibroblast phenotype and angiogenic properties. J. Pathol..

[24] Clavreul A., Etcheverry A., Tétaud C., Rousseau A., Avril T., Henry C., Mosser J., Menei P. (2015). Identification of two glioblastoma-associated stromal cell subtypes with different carcinogenic properties in histologically normal surgical margins. J. Neurooncol..

[25] He Z., You C., Zhao D. (2018). Long non-coding RNA UCA1/miR-182/PFKFB2 axis modulates glioblastoma-associated stromal cells-mediated glycolysis and invasion of glioma cells. Biochem. Biophys. Res. Commun..

[26] Ius T., Ciani Y., Ruaro M.E., Isola M., Sorrentino M., Bulfoni M., Candotti V., Correcig C., Bourkoula E., Manini I. (2018). An NF-κB signature predicts low-grade glioma prognosis: A precision medicine approach based on patient-derived stem cells. Neuro Oncol..

[27] Wang R., Chadalavada K., Wilshire J., Kowalik U., Hovinga K.E., Geber A., Fligelman B., Leversha M., Brennan C., Tabar V. (2010). Glioblastoma stem-like cells give rise to tumour endothelium. Nature.

[28] Najbauer J., Huszthy P.C., Barish M.E., Garcia E., Metz M.Z., Myers S.M., Gutova M., Frank R.T., Miletic H., Kendall S.E. (2012). Cellular host responses to gliomas. PLoS ONE.

[29] Dzobo K., Dandara C. (2020). Architecture of cancer-associated fibroblasts in tumor microenvironment: Mapping their origins, heterogeneity, and role in cancer therapy resistance. Omics J. Integr. Biol..

[30] Gomes E.D., Vieira de Castro J., Costa B.M., Salgado A.J. (2018). The impact of mesenchymal stem cells and their secretome as a treatment for gliomas. Biochimie.

[31] Parker Kerrigan B.C., Hossain A., Yamashita S., Lang F.F. (2018). Stem cell therapy of gliomas. Prog. Neurol. Surg..

[32] Roger M., Clavreul A., Venier-Julienne M.-C., Passirani C., Montero-Menei C., Menei P. (2011). The potential of combinations of drug-loaded nanoparticle systems and adult stem cells for glioma therapy. Biomaterials.

[33] Birnbaum T., Roider J., Schankin C.J., Padovan C.S., Schichor C., Goldbrunner R., Straube A. (2007). Malignant gliomas actively recruit bone marrow stromal cells by secreting angiogenic cytokines. J. Neurooncol..

[34] Nakamizo A., Marini F., Amano T., Khan A., Studeny M., Gumin J., Chen J., Hentschel S., Vecil G., Dembinski J. (2005). Human bone marrow-derived mesenchymal stem cells in the treatment of gliomas. Cancer Res..

[35] Pavon L.F., Sibov T.T., de Souza A.V., da Cruz E.F., Malheiros S.M.F., Cabral F.R., de Souza J.G., Boufleur P., de Oliveira D.M., de Toledo S.R.C. (2018). Tropism of mesenchymal stem cell toward CD133+ stem cell of glioblastoma in vitro and promote tumor proliferation in vivo. Stem Cell Res. Ther..

[36] Appaix F., Nissou M.-F., van der Sanden B., Dreyfus M., Berger F., Issartel J.-P., Wion D. (2014). Brain mesenchymal stem cells: The other stem cells of the brain?. World J. Stem Cells.

[37] Kang S.-G., Shinojima N., Hossain A., Gumin J., Yong R.L., Colman H., Marini F., Andreeff M., Lang F.F. (2010). Isolation and perivascular localization of mesenchymal stem cells from mouse brain. Neurosurgery.

[38] Paul G., Özen I., Christophersen N.S., Reinbothe T., Bengzon J., Visse E., Jansson K., Dannaeus K., Henriques-Oliveira C., Roybon L. (2012). The adult human brain harbors multipotent perivascular mesenchymal stem cells. PLoS ONE.

[39] Lin W., Huang L., Li Y., Fang B., Li G., Chen L., Xu L. (2019). Mesenchymal stem cells and cancer: Clinical challenges and opportunities. BioMed Res. Int..

[40] Roger M., Clavreul A., Sindji L., Chassevent A., Schiller P.C., Montero-Menei C.N., Menei P. (2012). In Vitro and in vivo interactions between glioma and marrow-isolated adult multilineage inducible (MIAMI) cells. Brain Res..

[41] Breznik B., Motaln H., Vittori M., Rotter A., Lah Turnšek T. (2017). Mesenchymal stem cells differentially affect the invasion of distinct glioblastoma cell lines. Oncotarget.

[42] Aquilanti E., Miller J., Santagata S., Cahill D.P., Brastianos P.K. (2018). Updates in prognostic markers for gliomas. Neuro Oncol..

[43] Montemurro N. (2020). Glioblastoma multiforme and genetic mutations: The issue is not over yet. An overview of the current literature. J. Neurol. Surg. Part Cent. Eur. Neurosurg..

[44] Hermida M.A., Kumar J.D., Schwarz D., Laverty K.G., Di Bartolo A., Ardron M., Bogomolnijs M., Clavreul A., Brennan P.M., Wiegand U.K. (2020). Three dimensional in vitro models of cancer: Bioprinting multilineage glioblastoma models. Adv. Biol. Regul..

[45] Autier L., Clavreul A., Cacicedo M.L., Franconi F., Sindji L., Rousseau A., Perrot R., Montero-Menei C.N., Castro G.R., Menei P. (2019). A new glioblastoma cell trap for implantation after surgical resection. Acta Biomater..

[46] Najberg M., Haji Mansor M., Boury F., Alvarez-Lorenzo C., Garcion E. (2019). Reversing the tumor target: Establishment of a tumor trap. Front. Pharmacol..

[47] van der Sanden B., Appaix F., Berger F., Selek L., Issartel J.-P., Wion D. (2013). Translation of the ecological trap concept to glioma therapy: The cancer cell trap concept. Future Oncol..

